# Welcome to our new Section Editors

**DOI:** 10.1002/1878-0261.12380

**Published:** 2018-09-30

**Authors:** Julio E. Celis

It is now 1 year and 8 months since *Molecular Oncology* became fully Open Access in 2017 under the FEBS Press platform and Wiley. Interestingly, the number of submissions remained comparable during 2016 and 2017, and we are delighted to report that so far in 2018, submissions have almost doubled as compared to the same period last year.

To tackle the increase in submissions, we recently appointed Section Editors in basic/preclinical research (http://https://febs.onlinelibrary.wiley.com/hub/journal/18780261/editor-profiles#MBoutros, Heidelberg, Germany; http://https://febs.onlinelibrary.wiley.com/hub/journal/18780261/editor-profiles#GCalin, Houston, USA; http://https://febs.onlinelibrary.wiley.com/hub/journal/18780261/editor-profiles#GMelino, Cambridge, UK) and in clinical research (http://https://febs.onlinelibrary.wiley.com/hub/journal/18780261/editor-profiles#MNilbert, Copenhagen, Denmark; http://https://febs.onlinelibrary.wiley.com/hub/journal/18780261/editor-profiles#KPantel, Hamburg, Germany). Their expertise and commitment will be essential to the future development of the Journal, and together with the Editor‐in‐Chief, they will apply the same rigorous standards of peer review to ensure that the journal keeps its high standing among cancer journals worldwide.

In addition, we have strengthened the Editorial office by appointing http://https://febs.onlinelibrary.wiley.com/hub/journal/18780261/journal-menu/contact-us as Editorial Associate to coordinate editorial matters. Moreover, we have put into operation significant changes to the Web site to facilitate access to critical information.

I want to take this opportunity to thank José Moreira, with whom I started the Journal in 2007, for his instrumental and countless contributions to the development of the Journal, in particular during the more difficult early years. José will remain at the Journal as Review Editor. I would also like to thank http://https://febs.onlinelibrary.wiley.com/hub/journal/18780261/journal-menu/contact-us, our Editorial Assistant, for her valuable support, and Mary Purton, Coordinator of http://https://febs.onlinelibrary.wiley.com/, for her continuous advice during the transition process.

Finally, I would like to convey my sincere thanks to the readers for their continuing support and take this opportunity to invite you to submit your research articles to *Molecular Oncology*.

## Section editors

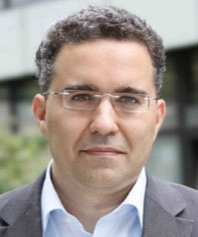

**Michael Boutros:** *genomics, functional genomics, oncogenic signaling, and target and drug discovery*.Michael Boutros is the head of the Division Signaling and Functional Genomics and Speaker of the Functional and Structural Genomics Program at the German Cancer Research Center (DKFZ). He is also Professor for Cell and Molecular Biology at Heidelberg University. After his PhD with Marek Mlodzik at the European Molecular Biology Laboratory (EMBL), he joined the laboratory of Norbert Perrimon at Harvard Medical School as a postdoctoral fellow. In 2003, he started his independent group at the DKFZ in Heidelberg funded by an Emmy‐Noether Grant of the German Research Foundation. He was also supported by the EMBO Young Investigator Program. He later became head of Division and full Professor at Heidelberg University. Michael Boutros’ research interests include functional genomic approaches to understand the regulation of cellular signaling in normal and cancer cells. His laboratory further develops and applies high‐throughput screening methodologies to dissect genetic networks and genotype‐specific vulnerabilities. He is supported by the European Research Council (ERC) and is an elected member of the European Molecular Biology Organization (EMBO).

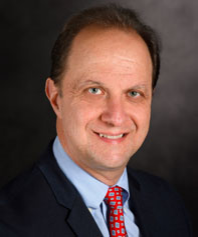

**George Calin:** *noncoding RNAs, cancer predisposition linked to noncoding RNAs, and new RNA therapeutic options for cancer patients*.George Adrian Calin received both his MD and PhD degrees at Carol Davila University of Medicine in Bucharest, Romania. After working in cytogenetics as undergraduate student with Dr. Dragos Stefanescu in Bucharest, he completed a cancer genomics training in Dr. Massimo Negrini's laboratory at University of Ferrara, Italy. In 2000, he became a postdoctoral fellow at Kimmel Cancer Center in Philadelphia, PA, and while working in Dr. Carlo Croce's laboratory, Dr. Calin was the first to discover the link between microRNAs and human cancers, a finding considered to be a milestone in microRNA research history. He is presently a Professor in the Experimental Therapeutics and Leukemia Departments at M. D. Anderson Cancer Center in Houston, where he studies the roles of microRNAs and other noncoding RNAs in cancer initiation and progression and in immune disorders, as well as the mechanisms of cancer predisposition linked to noncoding RNAs. Furthermore, he explores the roles of body fluid miRNAs as potential hormones and biomarkers, as well as new RNA therapeutic options for cancer patients.

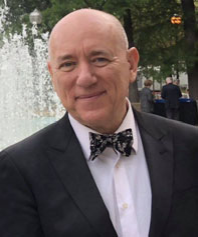

**Gerry Melino:** *cell death, DNA damage, p53, skin, and neuroectodermal tumors*.Gerry Melino, MD (Rome), PhD (London), Dr Sci hc (St Petersburg), is Head of the Genes versus Environment in DNA damage program at the Medical Research Council Toxicology Unit, UK, and Professor of Biochemistry, Faculty of Medicine, University of Rome‐Tor Vergata. His scientific interest, with over 500 papers (GS: 47103 citations, h‐index 96; Scopus: 31140 citations, h‐index 80), focuses upon programmed cell death in epidermal and neural models, and in particular on the family members of the tumor suppressor p53, namely p73 and p63. He identified splicing isoforms of p73 and p63, their involvement in DNA damage response, transcriptional regulation, degradation pathways, and mechanisms of death. His group has developed knockout mouse models to understand the biology of both p73 and p63. He previously worked on transglutaminases’ role in the formation of the cornified envelope in skin, creating mouse models, and contributing to the understanding of epidermal diseases.

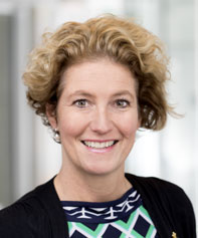

**Mef Nilbert:** *biomarkers and tumor genetics, molecularly targeted therapies, hereditary cancer syndromes, colorectal cancer, and sarcoma*.Mef Nilbert is a PhD in Tumor Genetics and an MD with specialization in oncology. Her clinical responsibilities have been focused on medical oncology, oncogenetics, gastrointestinal cancer, and sarcoma. As a professor of Oncology, she has developed research groups at the Lund University in Sweden and at the Copenhagen University in Denmark. Her research field is at the intersection of genomics, epidemiology, and clinical management, with applicability for refined diagnostics, risk prediction, and implementation of molecularly targeted cancer therapies. Current projects relate to risk modeling, molecular taxonomy, epidemiologic patterns, and predictive markers for immunotherapy in Lynch syndrome.

She has led a regional cancer center in Sweden with responsibilities for cancer registration, clinical guidelines, and political initiatives to improve modern cancer care. As part of this work, she has more recently contributed to research related to disease patterns, multidisciplinary treatment, and patients’ perspectives of cancer care.

Since 2017, she is the head of the Danish Cancer Society Research Center in Copenhagen, which is an international research environment with particular strengths in basic cancer biology and cancer epidemiology. The institute's scientific projects range from basic genomic repair to environmental risk factors and survivorship perspectives. As director of research, it is Prof. Nilbert's aim to further develop the institute's research agenda, grant high‐quality studies, support training of cancer researchers of tomorrow, and stimulate cross‐disciplinary scientific interaction and collaboration.

Professor Nilbert is also co‐editor of *Acta Oncologica* that has a more epidemiological–clinical focus in cancer research. She is now looking forward to contributing to *Molecular Oncology*, in particular with regard to molecularly targeted therapies.

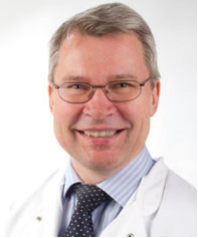

**Klaus Pantel:** *liquid biopsies, circulating tumor cells, cancer metastasis, solid tumors, and prognostic and predictive biomarkers*.Professor Pantel is Chairman of the Institute of Tumor Biology at the University Medical Center Hamburg‐Eppendorf. The institute is part of the Center for Experimental Medicine and the University Cancer Center Hamburg (UCCH). Professor Pantel graduated in 1986 from Cologne University in Germany and completed his thesis on mathematical modeling of hematopoiesis in 1987. After his postdoctoral period in the United States on hematopoietic stem cell regulation (Wayne State University, Detroit), he performed research at the Institute of Immunology, University of Munich, for 10 years. The pioneering work of Prof. Pantel in the field of cancer micrometastasis, circulating tumor cells, and circulating nucleic acids (ctDNA, microRNAs) is reflected by more than 400 publications in excellent high‐ranking biomedical and scientific journals (incl. NEJM, Lancet, Nature Journals, Cancer Cell, Science Translational Medicine, Cancer Discovery, PNAS, JCO, JNCI, and Cancer Res.), and Prof. Pantel was awarded the AACR Outstanding Investigator Award in 2010, German Cancer Award in 2010, and ERC Advanced Investigator Grant in 2011. Moreover, Prof. Pantel coordinates the European IMI consortium CANCER‐ID (http://www.cancer-id.eu) on blood‐based ‘Liquid Biopsies’ in lung and breast cancer, which comprises 37 partner institutions from academia, nonprofit organizations, and industry.

